# The relationship between social support and dietary salt choices among pregnant women in Hangzhou: a cross-sectional study

**DOI:** 10.3389/fpubh.2026.1783180

**Published:** 2026-06-01

**Authors:** Caifang Zheng, Tingting Zhao, Sujuan Zhu, Minjie Xu, Shuai Liu, Liangliang Huo

**Affiliations:** 1 Hangzhou Center for Disease Control and Prevention, Hangzhou Health Supervision Institution, Hangzhou, Zhejiang, China; 2 Zhejiang Key Laboratory of Multi-Omics in Infection and Immunity, Hangzhou, Zhejiang, China

**Keywords:** iodized salt, objective support, pregnant women, social support, subjective support

## Abstract

**Objective:**

To explore the relationship between pregnant women's social support level and iodized salt selection.

**Methods:**

In 2021, one survey site was randomly selected from each of the 13 counties and districts (cities) in Hangzhou. At each survey site, 100 pregnant women were selected to conduct face-to-face questionnaires on basic information, family salt consumption choices, and social support.

**Results:**

A total of 1,317 pregnant women were investigated, and the average total social support score was 41.2 ± 7.2. Among them, 1,216 (92.3%), 100 (7.6%) and 1(0.1%) pregnant women had high, moderate and low levels of total social support, respectively. In the past year, 940 women (71.4%) chose iodized salt, 105 women (8.0%) chose non-iodized salt, and 272 women (20.7%) were indifferent. There were statistical differences in objective support, subjective support, utilization of support, and total support among pregnant women of different salt selection types (*F* = 59.756, 19.540, 45.419, 53.831, *P* < 0.001). The total social support was the highest in the iodized salt group (42.4 ± 6.9 points) and the lowest was in the indifferent group (37.5 ± 7.3 points).

**Conclusions:**

The overall social support of pregnant women in Hangzhou is good, but some women still consume non-iodized salt. We should pay attention to the objective support and support utilization of pregnant women to improve the consumption of iodized salt and ensure the iodine nutrition level during pregnancy.

## Introduction

Globally, iodine deficiency disorders (IDD) remain a significant public health issue. It is estimated that approximately 1.88 billion people worldwide are at risk of iodine deficiency (1). To address this issue, the World Health Organization has recommended Universal Salt Iodization (USI) as the most cost-effective preventive strategy since 1993. Currently, about 88% of the global population has access to iodized salt, and over 120 countries have implemented related programs (2, 3). Although significant progress has been made in prevention and control efforts, the improvement of iodine nutrition among special groups such as pregnant women still lags behind that of the general population. The problem of insufficient iodine intake among pregnant women remains particularly prominent (4, 5).

Pregnant women are in a special physiological stage. Maternal iodine deficiency during pregnancy may seriously affect the growth and development of the fetus, and may even cause physical and intellectual development disorders of the newborn (6, 7). The consumption of iodized salt is related to multiple factors such as individual cognition, family environment, market conditions and policy support (8, 9). As a psychological and practical resource brought by the relationship between an individual and family, friends, neighbors, and others, good social support can effectively promote the physical and mental health of pregnant women (10–12). In terms of dietary behavior, the mechanism of the role of social support is worthy of attention. The informational support provided can influence dietary decisions and intentions, which in turn may affect the choice of iodized salt (13, 14). Pregnant women not only have to undergo physiological changes during pregnancy, but also bear the transformation of society, economy, family, and their roles (15, 16). To further understand the influencing factors of iodized salt consumption among pregnant women in Hangzhou and better guide pregnant women to scientifically supplement iodine, a survey on social support for pregnant women was conducted in 2021 to explore the relationship between social support and iodized salt choices.

## Participants and methods

### Participants

**Figure 1:**
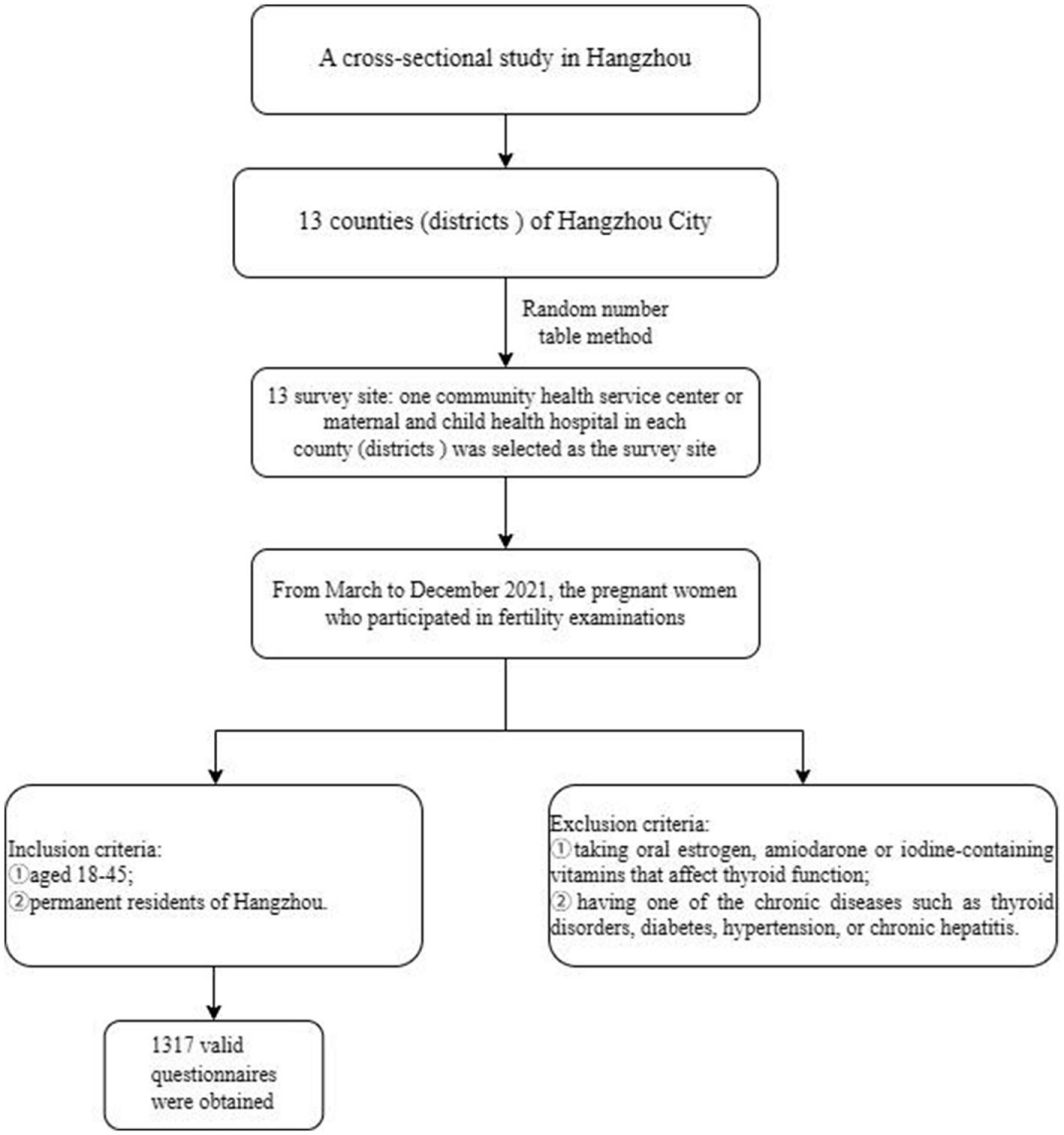
Flow diagram of participant selection process.

A cross-sectional survey study was conducted in Hangzhou in 2021. In February 2021, in 13 counties and districts (cities) of Hangzhou City, using the random number table method, namely Binjiang District, Chun'an County, Fuyang District, Gongshu District, Jiande City, Jianggan District, Lian'an District, Chongqing District, Tonglu County, Xihu District, Xiacheng District, Xiaoshan District and Yuhang District, one community health service center or maternal and child health hospital in each county and district (city) was randomly selected as the survey site according to the random number table method. From March to December 2021, no less than 100 pregnant women who participated in fertility examinations were selected at each site as the survey objects. Inclusion criteria: pregnant women who are permanent residents of Hangzhou, aged 18–45; Exclusion criteria: taking oral estrogen, amiodarone or iodine-containing vitamins that affect thyroid function; Having one of the chronic diseases such as thyroid disorders, diabetes, hypertension, or chronic hepatitis. Flow diagram of participant selection process was presented in Figure 1.

All procedures performed in studies involving human participants were approved by the Medical Ethics Committee of Hangzhou Center for Disease Control and Prevention (Ethics No. 2019–10). Informed consent was obtained from all individual participants included in the study.

### Measures

The questionnaire included demographic characteristics, the type of salt primarily used in the past year and social support (17). The type of salt included iodized salt, non-iodized salt, or indifferent. The term “indifferent” was defined as “respondents who reported no clear preference regarding household salt type or who had not actively paid attention to the type of salt used”. The social support score was with 10 items totaling 40 points and subjective support, objective support, support utilization, and total social support was calculated, respectively. This scale has been widely used among the Chinese population and has good reliability and validity (18). The scoring criteria for social support are shown in Table 1. Each survey site standardized the investigation process and obtained the informed consent of participants. The unified training investigator adopted a face-to-face questionnaire survey, and the questionnaire was considered valid if completed by the investigator.

**Table 1:** The criteria for social support scores.

Criteria	Objective support	Subjective support	Support utilization	Total support
Low level	< 10	< 16	< 6	< 20
Moderate level	10–15	16–24	6–9	20–30
High level	>15	>24	>9	30–40

### Statistical analysis

EpiData 3.0 software was used to establish a database for double entry of questionnaires, and SPSS 26.0 software was used for statistical analysis. Continuous variables were described using means and standard deviations (x̄ ± s), while categorical variables were described using frequencies and percentages (n (%)). One-way analysis of variance (ANOVA) was used for group comparisons. *P* < 0.05 were considered to be statistically significant.

## Results

### Basic characteristics

A total of 1,317 pregnant women were included (Table 2), with an average age of 35.2 ± 4.5 years. Most were first-time mothers (61.7%), 407 women (30.9%) in early pregnancy, 674 (51.2%) in mid-pregnancy, and 236 (17.9%) in late pregnancy. A majority (66.9%) were employed, and most had education levels of college or above (57.2%). Annual income ranged from 30,000 to 100,000 RMB for 61.8% of participants.

**Table 2:** Baseline characteristics of 1,317 participants.

Characteristics	*N*	Percentage (%)
Age (years)	1,317	35.2 ± 4.5
Nationality	
Han nationality	1,288	97.8
Other nationalities	29	2.2
Number of pregnancies	
1	812	61.7
≥2	505	38.3
Pregnancy (week)	
Early pregnancy (1–12 weeks)	407	30.9
Mid-pregnancy (13–27 weeks)	674	51.2
Late pregnancy (28–40 weeks)	236	17.9
Employment situation	
Employed	881	66.9
Unemployed	436	33.1
Education	
Junior college below	564	42.8
College degree or above	753	57.2
Annual income (RMB)	
< 3.0	145	11.0
3.0–6.0	396	30.1
6.0–10.0	418	31.7
≥10.0	358	27.2

### Social Support Scores

Among 1,317 pregnant women, the average total score of social support was 41.2 ± 7.2. 92.3% (1,216/1,317) were at a high level and 52.7% (694/1,317) of pregnant women had low objective support scores. Most of them had moderate (47.5%) and high (49.5%) subjective support scores, and 67.9% (894/1,317) had moderate utilization scores, as shown in Table 3.

**Table 3:** Social support scores of 1,317 participants.

Social support	Scores (x̄ ±s)	Low support *n* (%)	Moderate support *n* (%)	High support *n* (%)
Objective support	9.1 ± 2.7	694 (52.7)	611 (46.4)	12 (0.9)
Subjective support	24.2 ± 4.6	40 (3.0)	626 (47.5)	651 (49.5)
Support utilization	7.9 ± 2.0	140 (10.6)	894 (67.9)	283 (21.5)
Total support	41.2 ± 7.2	1 (0.1)	100 (7.6)	1,216 (92.3)

### Social support and salt choices

Of the pregnant women surveyed, 940 (71.4%) primarily chose iodized salt in the past year, 105 (8.0%) chose non-iodized salt, and 272 (20.7%) were indifferent. There were statistically significant differences in objective support, subjective support, support utilization, and total support scores among the different salt choice groups (*F* = 59.759, 19.540, 45.419, 53.831, *P* < 0.001). The iodized salt group had the highest total social support score (42.4 ± 6.9 points), while the indifferent group had the lowest (37.5 ± 7.3 points), as shown in Table 4.

**Table 4:** Social support scores with different salt choices.

Social support	Salt choices(x̄ ±s)			*F*	*P-*value	Partial η^2^
	Iodized salt	Non-iodized salt	Indifferent^*^			
Objective support	9.5 ± 2.4	9.4 ± 2.4	7.6 ± 3.2	59.756	< 0.001	0.083
Subjective support	24.7 ± 4.6	23.0 ± 4.6	23.0 ± 4.4	19.540	< 0.001	0.029
Support utilization	8.2 ± 1.9	8.0 ± 1.9	6.9 ± 1.9	45.419	< 0.001	0.065
Total support	42.4 ± 6.9	40.3 ± 6.5	37.5 ± 7.3	53.831	< 0.001	0.076

One-way analysis of variance (ANOVA) was used for group comparisons.

^*^“Indifferent” was defined as “respondents who reported no clear preference regarding household salt type or who had not actively paid attention to the type of salt used”.

## Discussion

Iodine deficiency in pregnant women not only affects their health but also may affect the mental and physical development of fetuses and newborns. Previous investigations have shown that the consumption rate of qualified iodized salt fluctuates, and iodine deficiency persists in the pregnant population in Zhejiang province (19, 20). The consumption of iodized salt is very important to ensure the iodine nutrition of pregnant women, and the lack of iodine-related knowledge is an important factor affecting the consumption of iodized salt (21). This survey reveals that 28.7% of pregnant women do not choose iodized salt. The reasons for this situation are various, which may be due to the factors of pregnant women themselves, such as the blind area of health education, insufficient knowledge of prevention and treatment of iodine deficiency diseases, and social factors, such as regional traditional diet structure and the continuous expansion of non-iodized salt sales channels in the market.

With the transformation of the bio-psycho-social medical model, the influence of social factors on pregnant women has attracted more and more attention. Numerous studies suggest that effective use of social support can significantly improve quality of life. As a special population, the influence of social support on pregnant women is different from that of the general population (22–24). Previous research on social support among pregnant women has focused on psychological mechanisms: without adequate social support, pregnant women may experience depressive symptoms due to the physiological changes and psychological challenges of motherhood. Relevant studies at home and abroad also prove that social support is closely related to mental health, and social support can improve individuals' self-confidence, ability to solve negative emotions, mental health level, and quality of life (25, 26). Nielsen et al. suggested that if effective social support from the family can be obtained in time, maternal depression can be avoided to the greatest extent (27).

Subjective social support refers to the emotional experience of being respected, supported, and understood within society, often evaluated through the support received from family members, colleagues, etc. This study found that half of surveyed women had moderate and high levels of subjective support. Previous studies have indicated that without the emotional support of close family or friends, pregnant women, especially first-time mothers, feel lonely and helpless (28, 29). The support from family members, especially spouses and grandparents, holds particular significance for healthy behaviors during pregnancy (30). Sokhamkaew et al. conducted a quasi-experimental study among pregnant women in Thailand, further confirming the crucial role of spouse support in improving iodine nutrition among pregnant women (31). Social support may indirectly influence a pregnant woman's dietary behavior by enhancing her self-regulation ability, providing empirical support for understanding the mechanism of social support within the Asian context (32). Social support is an important protective factor for ensuring mental health during pregnancy and promoting healthy behaviors (33). In this study, a considerable proportion of pregnant women were in the middle or low level of objective support scores. Objective support includes tangible support and intangible support. Tangible support mainly provides material assistance such as material economy and behavioral support. However, information and advice on pregnant women's health during pregnancy are intangible support and tend to be supported by guiding information (34). Raman S et al. found that pregnant women's health information support during pregnancy mainly comes from their caretakers, especially their mothers' suggestions (35), but the information support for pregnant women rarely mentions the guidance and suggestions from professional healthcare personnel (36). Otherwise, the comprehensive study by Al-Mutawtah M et al. suggested that information support could alleviate the stress and anxiety of pregnant women during pregnancy, and it was essential for pregnant women and their caregivers to obtain professional health guidance information (37). Therefore, improving the awareness rate of iodine deficiency diseases in pregnant women's social networks and focusing on strengthening publicity and popular science can effectively improve the awareness of scientific iodine supplementation for pregnant women and their caregivers and ensure the iodine nutrition level during pregnancy.

The average support utilization score among the pregnant women surveyed in this study indicated a moderate level. Rhonda Small et al. conducted a study on social support intervention for lactating women and found that although social support intervention measures focusing on health guidance were provided by professional healthcare personnel, the study subjects attached more importance to emotional support provided by family members or friends around them (38). When social support is available, pregnant women who perceive and actively utilize their social support networks are more likely to improve their overall social support levels.

Furthermore, 20.7% of the surveyed pregnant women in this study held an “indifferent” attitude toward the type of salt used in their households. This proportion is not to be ignored. Although the universal salt iodization policy has been implemented in China for several decades and significant progress has been made in the prevention and control of iodine deficiency disorders, the public's awareness of the importance of iodine nutrition may have shifted from “alert” to “habitual neglect” (39, 40). From the perspective of health information acquisition and cognition, the “indifferent” attitude may directly reflect the lack of effective information support in the social network of pregnant women. On the other hand, through precise health communication and enhancing authoritative information support from medical staff and families, it is entirely possible to turn this group of people into committed practitioners of scientific iodine supplementation.

This study has several limitations. Firstly, this study adopted a cross-sectional design. Although it revealed the association between social support and pregnant women's choice of iodized salt, as pointed out by the reviewers, this design itself limited our ability to make causal inferences. Secondly, we were unable to perform a multivariable analysis controlling for all potential confounders such as education, income, employment status, and pregnancy stage.

Overall, this study showed that the overall social support status of pregnant women in Hangzhou is relatively good, with subjective support scores at moderate to high levels. However, scores for objective support and support utilization remain at moderate to low levels. It is necessary to improve the awareness of scientific iodine supplementation for pregnant women and ensure the iodine nutrition level during pregnancy by improving the objective support and utilization of social support for pregnant women. The findings of this study have significant practical guiding significance for improving the iodine nutritional status of pregnant women and optimizing public health intervention strategies. Firstly, public health interventions should shift from the traditional “individual education” model to the “social network intervention” model, especially involving participatory health education by the main family caregivers such as spouses, mothers, and mothers-in-law. At the same time, emphasis should be placed on enhancing the accessibility and authority of information support, and fully leveraging the authoritative role of obstetricians, community maternal and child health workers, and midwives in the dissemination of iodine nutritional information, to make up for the professional shortcomings of family information support. Additionally, comprehensive support services that combine psychological counseling and behavioral guidance should be developed to help pregnant women convert the perceived social support into actual health behaviors, and improve the utilization rate of social support. Through the three-level linkage of hospitals, communities, and families, a full-process and comprehensive support for pregnant women's iodine nutrition should be formed, ultimately achieving the transformation from “awareness” to “behavior”, and effectively ensuring the health of mothers and infants.

## Data Availability

The raw data supporting the conclusions of this article will be made available by the authors, without undue reservation.
